# Supplementary material for: A fault-intrusion-tolerant system and deadline-aware algorithm for scheduling scientific workflow in the cloud
Source: PeerJ Comput Sci. 2021 Nov 2;7:e747. doi: 10.7717/peerj-cs.747 (PMC8576554; doi:10.7717/peerj-cs.747)
Supplement: Supplemental Information 6 [file peerj-cs-07-747-s006.zip › Myproject1.1/docs/org/cloudbus/cloudsim/power/models/PowerModelLinear.html]

PowerModelLinear


JavaScript is disabled on your browser.


- Overview
- Package
- Class
- Use
- Tree
- Deprecated
- Index
- Help

- Prev Class
- Next Class

- Frames
- No Frames

- All Classes

- Summary:
- Nested |
- Field |
- Constr |
- Method

- Detail:
- Field |
- Constr |
- Method


org.cloudbus.cloudsim.power.models

## Class PowerModelLinear

- java.lang.Object
- - org.cloudbus.cloudsim.power.models.PowerModelLinear

- All Implemented Interfaces:
  :   PowerModel

  ---

    

  ```
  public class PowerModelLinear
  extends java.lang.Object
  implements PowerModel
  ```

  The Class PowerModelLinear.
  If you are using any algorithms, policies or workload included in the power package, please cite
  the following paper:
  Anton Beloglazov, and Rajkumar Buyya, "Optimal Online Deterministic Algorithms and Adaptive
  Heuristics for Energy and Performance Efficient Dynamic Consolidation of Virtual Machines in
  Cloud Data Centers", Concurrency and Computation: Practice and Experience (CCPE), Volume 24,
  Issue 13, Pages: 1397-1420, John Wiley & Sons, Ltd, New York, USA, 2012

  Since:
  :   CloudSim Toolkit 2.0

- - ### Constructor Summary

    Constructors

    Constructor and Description

    `PowerModelLinear(double maxPower,
    double staticPowerPercent)`

    Instantiates a new linear power model.
  - ### Method Summary

    Methods

    | Modifier and Type | Method and Description |
    | --- | --- |
    | `protected double` | `getConstant()` Gets the constant. |
    | `protected double` | `getMaxPower()` Gets the max power. |
    | `double` | `getPower(double utilization)` Get power consumption by the utilization percentage according to the power model. |
    | `protected double` | `getStaticPower()` Gets the static power. |
    | `protected void` | `setConstant(double constant)` Sets the constant. |
    | `protected void` | `setMaxPower(double maxPower)` Sets the max power. |
    | `protected void` | `setStaticPower(double staticPower)` Sets the static power. |

    - ### Methods inherited from class java.lang.Object

      `clone, equals, finalize, getClass, hashCode, notify, notifyAll, toString, wait, wait, wait`

- - ### Constructor Detail


    - #### PowerModelLinear

      ```
      public PowerModelLinear(double maxPower,
                      double staticPowerPercent)
      ```

      Instantiates a new linear power model.

      Parameters:
      :   `maxPower` - the max power
      :   `staticPowerPercent` - the static power percent
  - ### Method Detail


    - #### getPower

      ```
      public double getPower(double utilization)
                      throws java.lang.IllegalArgumentException
      ```

      **Description copied from interface: `PowerModel`**

      Get power consumption by the utilization percentage according to the power model.

      **Specified by:**
      :   `getPower` in interface `PowerModel`

      Parameters:
      :   `utilization` - the utilization

      Returns:
      :   power consumption

      Throws:
      :   `java.lang.IllegalArgumentException` - the illegal argument exception


    - #### getMaxPower

      ```
      protected double getMaxPower()
      ```

      Gets the max power.

      Returns:
      :   the max power


    - #### setMaxPower

      ```
      protected void setMaxPower(double maxPower)
      ```

      Sets the max power.

      Parameters:
      :   `maxPower` - the new max power


    - #### getConstant

      ```
      protected double getConstant()
      ```

      Gets the constant.

      Returns:
      :   the constant


    - #### setConstant

      ```
      protected void setConstant(double constant)
      ```

      Sets the constant.

      Parameters:
      :   `constant` - the new constant


    - #### getStaticPower

      ```
      protected double getStaticPower()
      ```

      Gets the static power.

      Returns:
      :   the static power


    - #### setStaticPower

      ```
      protected void setStaticPower(double staticPower)
      ```

      Sets the static power.

      Parameters:
      :   `staticPower` - the new static power


- Overview
- Package
- Class
- Use
- Tree
- Deprecated
- Index
- Help

- Prev Class
- Next Class

- Frames
- No Frames

- All Classes

- Summary:
- Nested |
- Field |
- Constr |
- Method

- Detail:
- Field |
- Constr |
- Method
